# Complications in cochlear implant surgery: a comprehensive review

**DOI:** 10.25122/jml-2025-0009

**Published:** 2025-10

**Authors:** Cristian Mircea Neagoș, Bianca Gabriela Nenec, Adriana Neagoș, Anca Sin

**Affiliations:** 1 Doctoral School of Medicine and Pharmacy, George Emil Palade University of Medicine, Pharmacy, Science and Technology, Târgu Mureș, Romania; 2 Department of Otorhinolaryngology, Târgu Mureș Emergency County Hospital, Târgu Mureș, Romania; 3 Department of Otorhinolaryngology, George Emil Palade University of Medicine, Pharmacy, Science and Technology, Târgu Mureș, Romania; 4 Department of Molecular Biology, George Emil Palade University of Medicine, Pharmacy, Science and Technology, Târgu Mureș, Romania

**Keywords:** cochlear implant, Gusher Syndrome, inner ear malformations, intracochlear inflammation, foreign body reaction, intracochlear fibrosis, otitis media, implant extrusion

## Abstract

Cochlear implantation is an established surgical intervention for patients with severe to profound sensorineural hearing loss. Although technological advancements have improved surgical outcomes, complications can still arise, affecting both short- and long-term postoperative results. Identifying and managing these complications is very important for optimizing patient outcomes. This review examined the key complications associated with cochlear implantation, discussing their mechanisms, clinical implications, and management strategies based on current literature. A comprehensive literature review was conducted using relevant studies from PubMed and other scientific databases. Key topics include intraoperative complications such as cerebrospinal fluid (CSF) leakage and electrode misplacement, as well as postoperative complications like intracochlear fibrosis, foreign body reactions, vestibular dysfunction, device extrusion, and infections, including otitis media and cholesteatoma. Despite being a generally safe procedure, cochlear implantation presents a range of complications, with incidence rates varying between pediatric and adult populations. Preoperative imaging and patient selection have an essential role in minimizing intraoperative risks such as CSF leakage, particularly in cases with inner ear malformations. Postoperative complications, including electrode migration, fibrosis, and vestibular dysfunction, can impact hearing outcomes and quality of life. Furthermore, late complications such as chronic infections and device extrusion require long-term follow-up and, in some cases, revision surgery. Cochlear implantation is a highly effective auditory rehabilitation technique with a favorable safety profile. However, complications—ranging from minor surgical site infections to major device failures—necessitate ongoing clinical vigilance. Future advancements in electrode design, surgical techniques, and biocompatible materials hold promise for reducing complications and improving patient safety.

## Introduction

Cochlear implants provide auditory rehabilitation for patients with severe to profound sensorineural hearing loss. Advances in technology and surgical techniques have increased the number of eligible patients, including those with residual low-frequency hearing [1,2]. Successful cochlear implantation depends on multiple factors, including the etiology of hearing loss, the likelihood of achieving good postoperative outcomes, and the patient’s overall medical condition. In addition to surgical techniques and implant design, factors such as the experience of the surgical team, preoperative counseling, and postoperative care play essential roles in determining outcomes.

Surgeons must provide detailed information to patients and their families about the advantages of cochlear implantation, as well as its possible risks and complications [2,3]. Research indicates that complication rates associated with cochlear implants vary between adults and children. Common issues include intracochlear fibrosis, inflammatory reactions, and fluctuations in hearing performance, all of which necessitate ongoing monitoring and intervention [4-6]. Regardless of the implant type, an inflammatory response is commonly observed after surgery, leading to fibrosis, which can impair cochlear function and residual hearing over time. This inflammatory reaction is influenced by several factors, such as the design of the electrode, the material used in the implant, and the surgical technique [1]. To reduce the risk of surgical trauma and fibrosis, several strategies have been proposed, including improvements in biomaterials, modifications in electrode placement, and more refined surgical methods. Techniques such as sealing the cochleostomy with muscle tissue, minimizing trauma during posterior tympanotomy, and employing the round window approach for electrode placement are some approaches suggested to mitigate postoperative complications [7]. Gaining a deeper understanding of the cellular, anatomical, and molecular mechanisms involved in cochlear fibrosis and inflammatory responses is vital for enhancing the performance of future cochlear implants and ensuring better long-term auditory outcomes [1].

A thorough assessment of adverse events is crucial for enhancing both patient safety and the success of surgeries. Preventive measures, such as preoperative vaccination against pneumococcus, have been shown to reduce the risk of infections after surgery, which is an important consideration, especially for patients with inner ear abnormalities who are at an increased risk of complications [5]. Systematic autoimmune disorders may contribute to congenital hearing loss and adversely affect postoperative outcomes by promoting chronic inflammation and increasing the risk of long-term cochlear implant failure.

Postoperative outcomes are generally favorable, with only minor issues such as intraoperative fibrosis and changes in audiological performance being commonly reported [6]. In children, otitis media, and in adults, tinnitus and vestibular dysfunction are frequent minor complications. However, more severe issues, including implant migration, internal device failure, and the need for revision surgery, demand specialized management [8-10]. Age-related factors can also affect the rate of complications. Older individuals may face a higher risk of implant migration and postoperative dizziness, although studies indicate that when comorbidities are accounted for, complication rates are similar across age groups [11]. Additionally, the design and size of the implant’s external components play a critical role. Technological improvements have led to the creation of thinner implants and smaller incisions, reducing the likelihood of migrations. Postoperative complications, such as fibrosis within the cochlea, are influenced by factors like electrode thickness, insertion technique, speed, and alignment. The lateral wall approach increases the risk of cochlear damage and improper positioning, while the round window insertion and perimodiolar electrode arrays minimize surgical trauma and enhance auditory outcomes through better neural stimulation [11]. Advancements in cochlear implantation have provided substantial benefits for individuals with hearing loss. However, complications must be anticipated, tracked, and managed appropriately to ensure optimal long-term results. These complications are generally classified as intraoperative, early postoperative, or late postoperative (beyond 3 months after surgery), with each category requiring specific monitoring and intervention. Early detection and proper management are crucial for achieving a favorable prognosis. Additionally, preoperative counseling about potential risks and their management is an essential part of the cochlear implantation process [12].

This article reviews the current literature on the complications associated with cochlear implantation, focusing on risk factors, preventive measures, and therapeutic approaches.

## Material and Methods

A systematic literature review was conducted to examine the complications associated with cochlear implantation, including both intraoperative and postoperative adverse events. Relevant studies were identified through a structured search of PubMed and other scientific databases. The search strategy focused on identifying studies related to complications associated with cochlear implantation, including both intraoperative and postoperative adverse events. Studies were included if they were published in English and examined surgical or postoperative complications of cochlear implantation. Eligible sources included clinical trials, cohort studies, case reports, and systematic reviews. Studies without available full text, those focusing on non-surgical interventions, or articles not directly addressing cochlear implant complications were excluded.

Data were extracted from selected studies and categorized based on the nature of the reported complications. The review was structured into key areas: intraoperative complications, such as Gusher syndrome, electrode misplacement, and intraoperative cerebrospinal fluid (CSF) leakage; electrode insertion and positioning issues, including scalar translocation, tip fold-over, and electrode migration; intracochlear inflammatory responses and fibrosis, particularly foreign body reactions and cochlear fibrosis; vestibular dysfunction, including postoperative dizziness, balance disorders, and vestibular damage; late postoperative complications, such as implant extrusion, chronic infections, and device failure; and infectious complications, including otitis media, cholesteatoma, and postoperative meningitis.

Findings from the selected studies were synthesized to provide an evidence-based perspective on the incidence rates, risk factors, clinical manifestations, and management strategies for each complication. The discussion was structured to highlight current surgical and postoperative risk mitigation approaches. No meta-analysis was performed, as this review primarily focused on a qualitative synthesis of available literature rather than statistical aggregation of outcomes.

### Inner ear malformations and Gusher syndrome: intraoperative complication risks

Gusher syndrome is a rare intraoperative complication during cochlear implantation, where CSF leaks from the cochleostomy or round window membrane. This condition occurs in approximately 1 in 100 cochlear malformations, with an incidence of 40-50%. This is due to an existing connection between the internal auditory canal and the perilymph of the inner ear [13]. Preoperative imaging techniques such as computer tomography (CT) and magnetic resonance imaging (MRI) help identify cochlear abnormalities that might predispose patients to Gusher syndrome. However, in about 1.5% to 2.5% of cases, the syndrome can still occur even when the imaging appears normal. This is because certain small communications between the cochlea and the internal auditory canal may not be visible on these imaging scans [14].

Preoperative assessment should focus on evaluating the size of the cochlea, the width of the vestibular aqueduct, and the integrity of the modiolus, particularly when cochlear implantation is planned via the round window approach [13]. Inner ear malformations, especially those involving bony defects in the internal auditory canal (IAC) floor, are strongly associated with Gusher syndrome [15]. When intraoperative CSF leakage occurs, immediate measures such as administering intravenous mannitol, using propofol to reduce CSF pressure, and inserting electrodes rapidly are recommended. Additionally, there is a risk of electrode extrusion, which necessitates secure fixation of the electrode using periosteum or muscle [13]. Several surgical techniques have been proposed to manage intraoperative CSF leakage, including positioning the patient in Trendelenburg, controlled hyperventilation to reduce venous return, and promoting cerebral vasoconstriction through hypercapnia [13]. In some situations, complete blockage of the middle ear and Eustachian tube by performing subtotal petrosectomy may be required. It is crucial to note that Gusher syndrome is an intraoperative complication associated with detectable or non-detectable inner ear malformations, but it can also occur in cases of apparently normal ears [13]. Although cochlear implantation is considered a safe procedure, the complication rate can reach 12.5%, particularly in children [14].

The presence of inner ear malformations significantly increases the likelihood of intraoperative complications, with structural abnormalities such as Mondini dysplasia and common cavity deformities posing the highest risk for Gusher syndrome [15]. Evaluating surgical outcomes in these cases requires consideration of demographic, radiological, neurophysiological, and intraoperative factors. A conservative management approach is often recommended in the immediate postoperative period to minimize risks [16]. The literature reports that approximately 30% of Gusher syndrome cases are associated with inner ear malformations, primarily due to defects in the IAC floor. Therefore, much discussion revolves around cochlear implantation in cases of inner ear malformations and associated risks [17].

### Cochlear implantation considerations in inner ear malformations

Anticipating and managing complications in patients with inner ear malformations is essential for surgical planning and patient counseling. Cochlear implantation in malformed cochleae presents greater intraoperative risks, particularly in cases where the modiolus is absent or underdeveloped [17]. In incomplete partition type I (IP-I) and type III (IP-III) malformations, there is a significantly increased likelihood of CSF leakage. Gusher syndrome can manifest as low-flow or high-flow CSF leaks, occurring either through the cochleostomy or via a fistula at the stapes footplate. In cases where the modiolus or cochlear nerve canal is underdeveloped, there is also a risk of misplacement of the electrode array into the internal auditory canal or the vestibule, further complicating surgical outcomes [17]. The inner ear malformations at highest risk for these complications include common cavity deformities, incomplete partition type I (IP-I), and type III (IP-III) anomalies [18]. In contrast, the hypoplastic cochleae may cause incomplete CI insertion due to smaller dimensions. In general, if resistance is met, the array should be redirected or insertion stopped [17]. For patients with common cavity malformations or underdeveloped modiolus structures (IP-I, IP-III), the use of lateral wall electrodes is often preferred over perimodiolar arrays, as the latter can be challenging to position effectively in such cases [19]. A fully banded electrode design can be used to ensure stimulation in cases where the position of neural elements within the cochlea is inconsistent or occurs along the lateral wall. The shorter and thinner electrodes should be considered in cases of a hypoplastic cochlea [20].

### Postoperative management and outcomes

Managing postoperative care following intraoperative Gusher syndrome remains complex, as CSF leakage can still occur even in children with postlingual deafness and normal preoperative imaging. To avoid delayed complications, it is essential for surgeons to thoroughly seal the electrode insertion site and consider both surgical and non-surgical methods to control any CSF outflow [18]. Performing cochlear implantation in individuals with inner ear malformations presents distinct surgical difficulties, necessitating meticulous preoperative assessment and flexibility during the procedure. When imaging reveals structural abnormalities, it becomes critical to adapt the surgical approach to the patient’s specific anatomy. Intraoperative adjustments may be required, including choosing the most suitable electrode array and insertion technique to enhance hearing outcomes and reduce risks [19].

Roughly 20% of congenital hearing loss cases are linked to inner ear malformations, most commonly involving incomplete partition defects and cochlear hypoplasia. These anatomical issues pose two major risks during surgery: Gusher syndrome and anomalies of the facial nerve. Both must be assessed carefully in the preoperative stage [20]. Incomplete partition type I malformations, in particular, may be associated with a cochlear fistula, leading to a pronounced Gusher syndrome during surgery and heightened risk of meningitis [21]. Additionally, recent findings have identified Gusher syndrome in cases of superior semicircular canal dehiscence when the internal auditory canal is involved, further broadening the range of anatomical defects that can lead to CSF leakage.

Due to the intricate nature of cochlear implant procedures in malformed ears, such surgeries are best conducted in specialized centers with experience in managing cochlear anomalies [22]. Postoperative results in patients with cochlear-vestibular malformations are often less optimal, with many demonstrating limited ability to process and benefit from the auditory signals delivered by the implant [23].

### Electrode insertion and positioning complications

The literature highlights various complications related to electrode positioning in the cochlea and the management of incorrect electrode placement. Incomplete electrode insertion and twisting are more frequently observed with straight electrodes, although their overall frequency remains below 2% in cases of cochlear implantation in a normal cochlea. Electrode tip curvature occurs more commonly with perimodiolar electrodes compared to straight electrodes, but remains below 5% [24]. Electrode migration, however, is a significantly greater concern with straight electrodes, with reported migration rates reaching 46% [24]. This may be due to the lack of proximity to the modiolus, which increases the likelihood of post-insertion movement. Scalar translocation has been described in both types of electrodes, with a higher rate of 56% reported for perimodiolar electrodes inserted through cochleostomy, with reduced contact of the electrode with the cochlear lateral wall [25]. Scalar translocation causes cochlear trauma due to the electrode penetrating from the scala tympani into the scala vestibuli or the middle scala, negatively affecting postoperative audiological performance [25]. Studies indicate that scalar translocation and concomitant electrode tip curvature, which negatively affect postoperative results, are more frequent with perimodiolar electrodes compared to straight ones. This may explain why straight electrodes, with intimate contact with the cochlear wall, are preferred to prevent intracochlear trauma [25].

Electrode positioning complications account for a significant proportion of perioperative complications, impacting the benefits of post-implantation outcomes. These complications can be minimized through proper surgical planning, careful preoperative evaluation, and intraoperative imaging, which can help reduce the impact of faulty electrode positioning. The surgeon must anticipate the risks of incorrect electrode placement and adhere to proper intraoperative steps and timings. The type of electrode influences electrode positioning. Studies have shown that longer, flexible, straight electrodes, such as those measuring 31.5 mm with direct contact with the cochlear lateral wall, provide good placement regardless of the brand. The rate of electrode tip curvature is 5.3% for perimodiolar electrodes and 1% for straight electrodes [24].

The optimal surgical approach for electrode insertion remains a subject of discussion. Both round window and cochleostomy techniques can achieve correct insertion into the scala tympani when cochlear structures are well-visualized on preoperative imaging [26,27]. Studies suggest that insertion through the round window is associated with a perimodiolar electrode position, which places the electrode closer to the cochlea's neural substrate and shortens the electrical circuit. This results in increased potential for electrical transmission due to the reduced distance from the electrode to the modiolus. Intraoperative and postoperative measurements indicate that insertion through the round window offers several advantages over cochleostomy and is demonstrated to be much safer [26].

### Intracochlear inflammation, foreign body reaction, and intracochlear fibrosis: complications associated with cochlear implantation

Cochlear implantation is generally considered a safe and effective procedure, with complication rates ranging between 6% and 20% [27]. Major complications require surgical interventions, while minor ones can be treated with medication. Among the rarer but clinically significant complications are intracochlear inflammation, foreign body reactions, and fibrosis, which can negatively impact auditory outcomes and, in severe cases, necessitate implant removal. A comprehensive diagnostic approach is essential for identifying and managing intracochlear complications. This includes detailed patient history, immunological and autoimmune testing, electrophysiological evaluations, and high-resolution imaging [28].

### Intracochlear fibrosis and cochlear ossification

Cochlear ossification is not an absolute contraindication for cochlear implantation; instead, there are specific surgical guidelines and techniques designed to facilitate implantation in the presence of ossified cochlear structures [29]. Intracochlear fibrosis, although rare, can contribute to progressive hearing loss and pose challenges for cochlear implantation. The etiology of fibrosis is diverse, including infections, inflammations, and possible procedures following cochlear implantation [29]. Causes of ossification and scarring tissue growth in the cochlea can produce progressive cochlear obstruction. Imaging is crucial and sensitive for identifying fibrosis and cochlear ossification, and postoperative follow-up is also important for monitoring audiological outcomes. The causes of cochlear ossification and scar tissue growth will determine the progressive obstruction of the cochlea. These can be identified by high-resolution computed tomography and magnetic resonance imaging. The literature, however, presents only a few and selected cases of cochlear fibrosis. In the preoperative preparation of patients, the degree of cochlear ossification and fibrosis should be considered, but it can sometimes go unnoticed in audiometric tests. When opening the cochlea through the round window, the surgeon can identify the presence of an ossified structure, the tympanic ramp through which the cochlear implant is inserted [30]. Postoperative audiometric evaluation is important to establish audiological results.

### Foreign body reaction and biocompatibility issues

The foreign body response to cochlear implant electrode materials can significantly impact device functionality, battery longevity, and residual hearing preservation. This immune-mediated reaction may lead to chronic inflammation, fibrosis, and alterations in electrical conduction, ultimately reducing implant performance. The biocompatibility of the electrode material is essential in minimizing these adverse effects, as inadequate compatibility can result in electrode extrusion, persistent inflammation, and tissue damage.

The administration of dexamethasone has been shown to mitigate hearing loss caused by trauma during electrode insertion, as demonstrated in animal studies [29]. Additionally, in some cases, granulomatous reactions, eosinophilic infiltration, and localized inflammatory responses have been observed, particularly near the cochleostomy site, with statistically significant differences (*P* < 0.05) compared to distal regions [30].

When discussing cochlear implants and their complications, the foreign body reaction to implanted materials must be considered. For cochlear implants, the presence of such a reaction can significantly reduce the device's performance, battery life, and preservation of residual hearing [31]. The foreign body reaction is considered one of the most frequent and severe complications, which can lead to skin infections and subsequently implant extrusion [32].

### Vestibular dysfunction following cochlear implants

Vestibular issues are a known risk associated with cochlear implant procedures, with their occurrence affected by individual anatomy, surgical techniques, and patient-specific characteristics. Individuals with inner ear deformities, especially those with conditions like enlarged vestibular aqueduct (EVA) or incomplete partition type II (IP-II), are more prone to experiencing balance problems such as vertigo after surgery [32]. Intraoperative complications, such as Gusher syndrome, may also contribute to post-surgical instability in these patients. While malformations of the vestibule and semicircular canals are commonly linked to balance dysfunction, factors like the choice of electrode, method of insertion, and other post-surgical issues appear to have a limited impact on vestibular loss rates [33]. Evaluating vestibular function in children poses challenges, often allowing only partial testing. Even so, such assessments are helpful in pinpointing which ear was implanted and detecting any resulting vestibular damage [34].

Standard methods for evaluating vestibular function before and after cochlear implantation include caloric testing, video head impulse testing (vHIT), and vestibular-evoked myogenic potentials, both cervical (cVEMP) and ocular (oVEMP). These diagnostic tools are valuable for monitoring vestibular function over time, with follow-up testing often occurring around nine months after surgery [35-37]. In individuals with bilateral vestibular dysfunction, assessments of postural stability are particularly important for evaluating balance. Reduced or absent vestibular responses observed in caloric or rotational testing can play a significant role in determining surgical plans [38]. If a patient shows uneven vestibular function before surgery, surgeons typically choose to avoid implanting the ear with the stronger vestibular input in order to help preserve spatial orientation and balance. Reports on the incidence of vertigo after cochlear implantation vary significantly, with prevalence ranging from 0.33% to 75%, and vestibular impairment occurring in approximately 20-75% of cases [39]. Despite this wide range, long-term vestibular issues after surgery tend to be relatively uncommon. Notably, patients who already have vestibular dysfunction prior to the procedure do not necessarily face a higher likelihood of experiencing vertigo afterward, indicating that preoperative vestibular assessments may not always be predictive of post-implantation balance problems [39].

In cases of simultaneous bilateral implantation, utricular vestibular function is usually preserved. Cochlear implantation tends to affect saccular function more frequently. Persistent dizziness following implantation can significantly impact a patient’s quality of life. Clinical studies focus on assessing dizziness after cochlear implant surgery and the patient's quality of life using questionnaires. Results indicate that these questionnaires are a valid tool for documenting and evaluating dizziness that can affect quality of life. These can be used complementarily to assess peripheral vestibular dysfunctions [40].

### Tardive postoperative complications: wound injuries and cochlear implant extrusion

Extrusion of the cochlear implant is a rare but significant late postoperative complication with multiple underlying causes [41]. Effective management of cochlear implant extrusion demands the surgeon's expertise and persistence, with a focus on optimizing implant functionality and ensuring patient safety [42]. One of the most common causes of cochlear implant extrusion is damage to the external auditory canal, which may occur during the surgical procedure. Specifically, the external auditory canal can become very thin during surgery, thus increasing the risk of rupture. Additionally, if the electrode is curved within the mastoid, it can exert additional pressure on the posterior-superior wall of the external auditory canal. In pediatric cases, mastoid growth also plays a crucial role in the evolution of cochlear implantation outcomes [43].

Although electrode exposure is not a frequent complication, it requires meticulous management, including careful monitoring and assessment of implant functionality [44]. The exposure of the electrode post-cochlear implantation is a rare late complication, potentially resulting from implant migration or damage to the tympanic membrane. Both the surgeon and audiologist need to be aware of this issue, and surgical intervention to close the external auditory canal may be necessary [45]. In cases where electrode extrusion or exposure occurs, explantation might be required, although it is not always necessary if no associated infection is present [46]. The need for explantation should be assessed based on the presence of infection and the impact on the patient’s hearing and overall health.

Complications related to skin, including those reported in the literature, are relatively low, though they are more common in adults compared to children. Studies have documented skin complications and their impact on patients, emphasizing the importance of appropriate management and follow-up care [47]. Preoperative and postoperative antibiotic treatments are critical in preventing postoperative skin infections associated with cochlear implantation. These treatments help minimize the risk of infection and support overall surgical success. Proper antibiotic use is part of a broader strategy to prevent complications and ensure the long-term functionality and safety of the cochlear implant. Although extrusion and electrode exposure are rare complications of cochlear implantation, they require careful management and, in some cases, surgical intervention. Monitoring, preventive measures, and appropriate use of antibiotics play crucial roles in managing these complications and ensuring successful outcomes for patients undergoing cochlear implantation.

### Otitis media and cholesteatoma as complications of cochlear implantation

Chronic otitis media (COM) can negatively influence cochlear implant success by increasing the likelihood of infections following surgery. Therefore, it is essential to stabilize the COM prior to implantation to reduce these complications. In cases where infections arise after the procedure, a comprehensive approach should be taken, prioritizing implant preservation, managing recurring infections, and, if needed, closing off the mastoid cavity to control the problem [48]. Another important postoperative concern is acute mastoiditis, especially in pediatric patients who have undergone cochlear implantation. In rare cases, cochlear implantation may be associated with complications such as facial nerve stimulation, device migration or failure, postoperative infection, and CSF leakage. Moreover, inflammatory responses can lead to intracochlear fibrosis and ossification, potentially affecting implant function and auditory outcomes. Though uncommon, these complications underscore the importance of careful patient selection, surgical technique, and postoperative monitoring. Some studies suggest that removing the implant may be necessary in these scenarios [48,49]. Subperiosteal abscesses have been reported in roughly 14.3% of affected individuals [49]. Initial management typically involves intravenous antibiotics, but surgical treatment may be required to avoid more serious issues such as implant exposure or loss. The elevated incidence of these complications is partly attributed to the mastoidectomy performed during implantation, which, while standard, may raise the risk of such infections. Timely intervention is critical, regardless of how long it has been since the surgery, and in some cases, placing ventilation tubes may also be considered [49].

Acute otitis media and mastoiditis are among the most frequently encountered complications following cochlear implantation. Postoperative treatment often involves the administration of cephalosporins and the placement of ventilation tubes, particularly in pediatric patients. Special attention is required for children under four years of age, as they are more vulnerable to middle ear infections due to their immature Eustachian tube function and increased exposure to upper respiratory infections [50].

Although rare, cholesteatoma is a significant late-onset complication that can arise following cochlear implantation. Its development is sometimes associated with extensive drilling during surgery, which can compromise the bone structure of the external auditory canal and the tympanic ring. This structural weakening can result in altered pressure dynamics, potentially leading to cholesteatoma formation. If not addressed promptly, it may damage surrounding tissues, destabilize the implant, and harm the electrode array. Ongoing follow-up is especially important for patients with prior middle ear disease, as they may be more prone to this issue [51]. While cholesteatoma is infrequent in adults, it tends to appear earlier and more often in pediatric patients. Management often involves a subtotal petrosectomy, removal of the existing implant, and, when suitable, immediate reimplantation [52].

In cases where cochlear implantation is performed in the presence of chronic otitis media, two primary surgical principles are essential for long-term success. The first is achieving full control over chronic ear discharge before surgery, which involves eliminating active infection and ensuring that the middle ear mucosa is healthy and stable. The second principle involves preventing the spread of infection after implantation. This may require occluding the Eustachian tube and filling the mastoid cavity to reduce the likelihood of reinfection. In some cases, staged surgical approaches and careful removal of diseased mucosa are used to improve outcomes. However, keeping the mastoid cavity open can aid in clinical monitoring, even though it may raise the chances of implant-related complications such as electrode extrusion [53].

Otitis media and cholesteatoma are serious considerations in the management of cochlear implantation. Chronic otitis media requires thorough preoperative and postoperative care to minimize infection risks, while cholesteatoma, though rare, necessitates vigilant monitoring and specific treatment strategies to protect the implant and ensure successful outcomes.

## Conclusion

The analysis of several studies on cochlear implantation complications demonstrates that, although cochlear implants are recognized as relatively simple interventions, they carry a series of postoperative risks. Postoperative complications, although considered quite rare, are related not necessarily to the surgical technique but rather to the risk of postoperative infections (whether cutaneous or mastoid), in addition to foreign body reactions and implant damage with the risk of extrusion. Summarizing everything in one sentence, we can affirm that the surgical limits and long-term evolution of cochlear implants cannot be concretely established, even though this intervention is considered safe in the medium and long term. Cochlear implantation is a well-established and successful treatment option for patients with severe to profound sensorineural hearing loss. However, surgical complications still occur around the complex procedures performed in otolaryngology (ENT), which can range from minor issues to major life-threatening conditions affecting both functional (loss of CI function, CSF leak) and non-functional (bacterial infection or incision inflammation) output. This review aimed to summarize the current knowledge on reducing these challenges and explore ways forward towards an optimal approach.

Given that cochlear implants represent the most modern surgical technique for auditory rehabilitation in adults and children, with significant implications for quality of life, there is a tendency to extend surgical indications in the future, particularly to inform doctors in related specialties about the benefits of this medical device. The improvement in the quality of life for patients with cochlear implants will simultaneously increase the number of surgical interventions, leading to a rise in the rate of complications. In this context, surgeons must be knowledgeable about cochlear implantation methods and the techniques for managing complications that can arise immediately postoperatively, as well as in the medium and long term.
